# Infection Control Measures and Prevalence of SARS-CoV-2 IgG among 4,554 University Hospital Employees, Munich, Germany

**DOI:** 10.3201/eid2803.204436

**Published:** 2022-03

**Authors:** Johanna Erber, Verena Kappler, Bernhard Haller, Hrvoje Mijočević, Ana Galhoz, Clarissa Prazeres da Costa, Friedemann Gebhardt, Natalia Graf, Dieter Hoffmann, Markus Thaler, Elke Lorenz, Hedwig Roggendorf, Florian Kohlmayer, Andreas Henkel, Michael P. Menden, Jürgen Ruland, Christoph D. Spinner, Ulrike Protzer, Percy Knolle, Paul Lingor, on behalf of the SeCoMRI Study Group

**Affiliations:** University Hospital rechts der Isar, Munich, Germany (J. Erber, V. Kappler, B. Haller, H. Mijočević, C. Prazeres da Costa, F. Gebhardt, N. Graf, D. Hoffmann, M. Thaler, H. Roggendorf, F. Kohlmayer, A. Henkel, J. Ruland, C.D. Spinner, U. Protzer, P. Knolle, P. Lingor);; German Center for Infection Research, Munich (J. Erber, D. Hoffmann, J. Ruland, U. Protzer, C.D. Spinner);; Helmholtz Zentrum München-German, Neuherberg, Germany (A. Galhoz, M.P. Menden);; Ludwig-Maximilians University Munich, Martinsried, Germany (A. Galhoz, M.P. Menden);; German Center for Diabetes Research, Neuherberg (M.P. Menden);; Technical University of Munich, Munich (M.P. Menden)

**Keywords:** COVID-19, coronavirus disease, severe acute respiratory syndrome coronavirus 2, SARS-CoV-2, coronaviruses, viruses, respiratory infections, strategies, infection control measures, IgG, antibodies, university hospital employees, seroprevalence, healthcare workers, zoonoses, Munich, Germany

## Abstract

Hospital staff are at high risk for severe acute respiratory syndrome coronavirus 2 (SARS-CoV-2) infection during the coronavirus disease (COVID-19) pandemic. This cross-sectional study aimed to determine the prevalence of SARS-CoV-2 infection in hospital staff at the University Hospital rechts der Isar in Munich, Germany, and identify modulating factors. Overall seroprevalence of SARS-CoV-2-IgG in 4,554 participants was 2.4%. Staff engaged in direct patient care, including those working in COVID-19 units, had a similar probability of being seropositive as non–patient-facing staff. Increased probability of infection was observed in staff reporting interactions with SARS-CoV-2‒infected coworkers or private contacts or exposure to COVID-19 patients without appropriate personal protective equipment. Analysis of spatiotemporal trajectories identified that distinct hotspots for SARS-CoV-2‒positive staff and patients only partially overlap. Patient-facing work in a healthcare facility during the SARS-CoV-2 pandemic might be safe as long as adequate personal protective equipment is used and infection prevention practices are followed inside and outside the hospital

Healthcare workers (HCWs) are exposed to severe acute respiratory syndrome coronavirus 2 (SARS-CoV-2) in the private context, as well as professionally with varying exposure risk depending on their workplace. Prevalence rates have been measured as high as 13.7% in the New York, NY, USA, area, 10.2% in a nationwide study in Spain, 7.5% for 580 HCWs in a hospital in Spain, 6.4% for >3,000 HCWs in a tertiary hospital in Belgium, 4.0% for >2,8790 HCWs in Denmark, and 0.4%‒3.8% for hospitals in China (*1**–**6*). Working in coronavirus disease (COVID-19)‒designated units has been reported to carry an increased risk for infection (*4**,**7*).

The greater Munich area in Germany became the epicenter of a SARS-CoV-2 outbreak after a confirmed case was reported on January 27, 2020. A rapid and massive increase in SARS-CoV-2 infections occurred during March 2020, when infected persons returned from skiing resorts, such as Ischgl, Austria, where the spread of infection was dramatic (*8*). The University Hospital Munich rechts der Isar faced the challenge of rapidly increasing numbers of COVID-19 patients, combined with an increasing number of staff in quarantine. To reduce the spread of infections, guidelines for the use of personal protective equipment (PPE) for staff and patients were introduced, including the obligation to wear face masks in all areas of the hospital (Figure 1). In addition, a telephone hotline was established to provide staff with guidance for reverse transcription PCR (RT-PCR) testing and quarantine policies.

**Figure 1:**
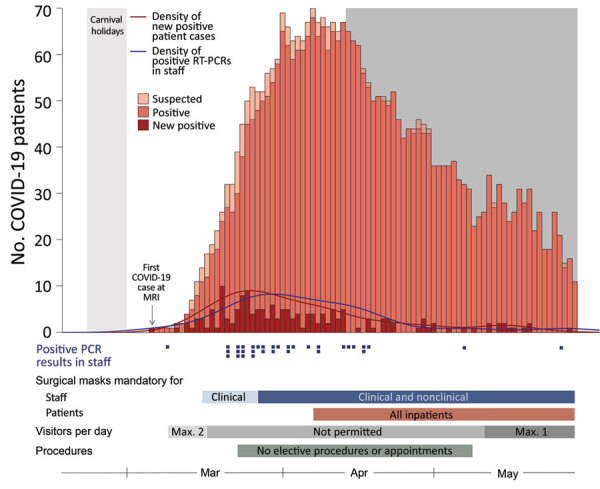
Prevalence and distribution of severe acute respiratory syndrome coronavirus 2 (SARS-CoV-2) infections in patients and staff at a university hospital in Munich, Germany. Shown is the number of all COVID-19 patients admitted to the hospital; the first COVID-19 patient was admitted on March 6, 2020. Light gray shading indicates dates of carnival holidays (February 22‒March 1, 2020); dark gray shading indicates dates of seroprevalence study (April 14‒May 29, 2020). Blue squares below graph indicate positive RT-PCR test results for SARS-CoV-2 RNA in university hospital staff. Bars below graph indicate densities of positive RT-PCR test results in staff (blue), new COVID-19 cases in patients (red), and limitations on number of visitors allowed and elective procedures and appointments (gray). COVID-19, coronavirus disease; Max., maximum; MRI, University Hospital Munich Rechts der Isar; RT-PCR, reverse transcription PCR.

To determine the epidemiology and immune response to SARS-CoV-2 and to identify best-practice approaches protecting staff and patients, we initiated a prospective, observational cohort study. The purpose of this study was to assess risk factors and evidence for infection, including clinical symptoms, and to determine the seroprevalence of SARS-CoV-2 antibodies.

## Materials and Methods

### Study Design and Participants

During April 14‒May 29, 2020, all clinical and nonclinical Munich rechts der Isar staff >18 years of age (n = 6,305) and medical students at the Technical University of Munich (n = 1,699) were invited to participate in this prospective, monocentric, observational study (Appendix Figure 1). Previous positive SARS-CoV-2 antibody tests results was not an exclusion criterion. Upon receiving written informed consent, we obtained demographic data; chronic medical conditions; occupation; work location; use of PPE; exposure to SARS-CoV-2‒positive patients, co-workers, or private contacts; symptom history; previous PCR testing for SARS-CoV-2; and outcome by using a standardized electronic questionnaire (Appendix) before the blood test result was known. We defined endoscopy, bronchoscopy, tracheal intubation, noninvasive ventilation, and transesophageal echocardiography as aerosol-generating medical procedures (AGPs).

We collected serum samples and subjected them to SARS-CoV-2 IgG and IgM testing (primary outcome). We tested for IgM in all persons until May 4, 2020 (n = 1,620), and thereafter only if IgG was positive or typical symptoms of COVID-19 were reported (n = 88) (Appendix Figure 2). Staff reporting symptoms or testing positive for IgM were recommended to undergo testing of nasopharyngeal swab specimens for SARS-CoV-2 by RT-PCR to exclude persistent infection. We stored personal data in a pseudonymized manner by using the open-source electronic case form system m4 DIS (BitCare GmbH, https://www.bitcare.de) (*9*). The study was approved by the Ethics Committee of the Technical University of Munich School of Medicine (approval no. 216/20S).

Since March 2020, a continuous infection surveillance program for all staff has been implemented at the University Hospital rechts der Isar in Munich, including an employee testing center and staff counselling (Corona Hotline), which is available 7 days per week. Persons who have symptoms compatible with COVID-19 or previous risk contacts are scheduled for testing for SARS-CoV-2 by RT-PCR from combined oropharyngeal and nasopharyngeal swab specimens. We included RT-PCR results of staff testing in the analysis if the study participants consented.

### Laboratory Analysis

We detected serum IgM and IgG against SARS-CoV-2 spike 1 protein or nucleocapsid protein by using a paramagnetic particle chemiluminescent immunoassay on an iFlash 1800 Immunoassay Analyzer (Shenzhen Yhlo Biotech Co., http://en.szyhlo.com). We subjected all serum samples that were positive for IgM or IgG (>10 AU/mL), all serum samples that had 5–10 AU/mL of IgG, and all serum samples from SARS-CoV-2 RT-PCR‒positive persons to confirmatory testing. For confirmation, we determined total antibodies against SARS-CoV-2 nucleocapsid by using an electrochemiluminescent immunoassay on a Cobas e411 Analyzer (Roche Diagnostics, https://www.roche.com). For all samples that had incongruent results, we determined IgG against SARS-CoV-2 spike 1 protein by using an ELISA (Euroimmun, https://www.euroimmun.com) and used immunoblotting to differentiate antibodies against nucleocapsid protein, spike 1 protein 1, and the receptor-binding domain of SARS-CoV-2 from those against seasonal coronaviruses (Mikrogen, https://www.mikrogen.de) (Appendix).

We extracted nucleic acids from nasopharyngeal swab specimens by using the mSample Preparation System DNA Kit identical to the Promega Maxwell Viral Total Nucleic Acid Extraction Kit (Promega, https://www.promega.com) according to a standard protocol on an m2000sp Device for RNA and DNA Extraction (Abbott, https://www.abbott.com). SARS-CoV-2 RT-PCR was performed by using SARS-CoV-2_N1 and SARS-CoV-2_N2 primer and probe sets for amplification on an ABI 7500 Device (Thermofisher Scientific, https://www.thermofisher.com) according to the protocol of the Centers for Disease Control and Prevention (Atlanta, GA, USA), as approved by the US Food and Drug Administration.

### Analysis of Patient and Staff Trajectories

We extracted anonymized patient mobility trajectory data from our hospital information system. COVID-19 was diagnosed when patients either showed typical clinical symptoms or had COVID-19‒typical findings in low-dose lung computed tomography scans and tested positive for SARS-CoV-2 by RT-PCR or for SARS-CoV-2 IgM or IgG (*10*). For spatiotemporal analysis of patient data, we used all trajectories available during December 30, 2019‒May 29, 2020, for each admitted COVID-19 patient because we could not determine the exact interval when the patients were contagious. We obtained trajectories of SARS-CoV-2 IgG seropositive staff from our questionnaire data if available (February 1‒May 29, 2020).

On the basis of the spatiotemporal trajectories of patients and staff, we created 2 types of representations: static representations over all timeframes and dynamically animated representations. The static representation is based on the relative proportion of patients or staff members at each hospital location normalized by all locations of the available trajectory time. For the dynamic representation, we illustrated 2 different relative proportions normalized by all time frames: the relative proportion of individual patients in each hospital location and the relative proportion of staff members mapped to their past locations for 14 days before they tested positive for SARS-CoV-2 IgG or were quarantined.

To analyze patient mobility within the hospital during the pandemic, we compared the spatial trajectories of COVID-19 patients to all patients given a diagnosis of any non‒COVID-19 pneumonia (viral or bacterial) during December 1, 2019‒June 10, 2020 (Appendix Figure 3). We performed all analyses by using R software version 3.6.0 (R Foundation for Statistical Computing, https://www.r-project.org) and made the source code available on GitHub (https://github.com/AnaGalhoz37/SeCOMRI).

### Statistical Analysis

Absolute and relative frequencies of positive tests for SARS-CoV-2 IgG and IgM (CLIA, Yhlo) are given for all study participants and relevant subgroups, along with exact 95% CIs for the estimated seroprevalence. To evaluate the association with potential risk factors, we estimated odds ratios (ORs) and corresponding exact 95% CIs (mid-p intervals). The distributions of antibody titers are visualized by boxplots or dot plots and are described by medians and quartiles. We used the Spearman rank correlation coefficient to evaluate the association between the time of IgG testing and the IgG titer. We did not adjust the 95% CI widths for multiplicity. Missing data were not imputed, and the number of missing values is presented for each variable. We conducted statistical analyses by using R software version 4.0.2 (R Foundation for Statistical Computing).

## Results

### Seroprevalence of SARS-CoV-2 IgG for 4,554 Hospital Employees

The study participation rate was 63.5% (4,001/6,305) for employees and 35.5% (603/1,699) for medical students; complete data for 4,554 persons were available for primary analysis (Appendix Figure 1). The mean age of the study participants was 38.5 years; 3,207 (70.4%) were women and 1,342 (29.5%) were men (Appendix Figure 4). Positive results for SARS-CoV-2 IgG were found for 108/4,554 study participants. For 102 persons, additional assays confirmed the SARS-CoV-2 IgG screening result (Appendix Table 1). Two additional persons who had a positive PCR result seroconverted during follow-up. Four persons who had IgG titers of 5–10 AU/mL in the screening assay, which is below the cutoff, were found to be positive in >2 other assays (Appendix Tables 1, 2). For 5 persons, the screening result could not be confirmed by the other assays used; for 1 person, there was insufficient material available to complete testing (Appendix Tables 3, 4). When we considered all 108 study participants who were positive for SARS-CoV-2 IgG in >2 different assays, we determined a seroprevalence of 2.4% (95% CI 1.9%–2.9%) (primary endpoint).

### Individual and Occupational Risk Factors for SARS-CoV-2 Infection

**Figure 2:**
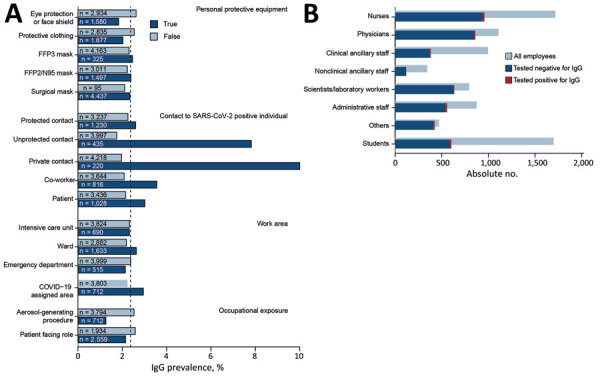
Prevalence of severe acute respiratory syndrome coronavirus 2 (SARS-CoV-2) IgG by risk factor and occupational group among staff members at a university hospital, Munich, Germany. A) Risk factors by category. Vertical dashed line indicates overall seroprevalence of 2.4%. B) Occupational groups. COVID-19, coronavirus disease; FFP, filtering face piece.

The first patient who had PCR-confirmed COVID-19 was admitted to our university hospital on March 6, 2020, and 163 COVID-19 patients were hospitalized during March 6‒May 29 (Figure 1). Infection prevention measures, such as the obligation to wear surgical masks, physical distancing measures, visitor rules, or policies for nonurgent procedures, were dynamically adjusted according to the prevalent pandemic situation (Figure 1). Risk factors for infection of staff were identified through correlation of self-reported survey data with seropositivity for SARS-CoV-2 IgG (Table 2). We found an association between seropositivity and male sex (OR 1.54, 95% CI 1.03–2.27) or age; the highest frequency was observed for persons 51–60 years of age (OR 1.75, 95% CI 1.06–2.85, compared with persons <30 years of age) (Table 1; Appendix Figures 4, 5). We found a higher relative frequency of seropositivity for persons who had diabetes mellitus (OR 2.96, 95% CI 1.01–6.81) but observed no major differences in staff who had preexisting pulmonary or cardiovascular disease (Table 2; Appendix Figure 5). Seropositivity was decreased for smokers (OR 0.52, 95% CI 0.26–0.94) (Table 2); relevant difference in seropositivity was observed between HCWs involved in direct patient care, including care of COVID-19 patients, and HCWs working in intensive care units or the emergency department compared with staff members not working in these units and not performing patient-associated tasks (Table 2; Figure 2, panel A; Appendix Figure 5).

**Table 2:** SARS-CoV-2 seroprevalence for healthcare workers, by self-reported risk factors and symptoms, at a university hospital, Munich, Germany*

Characteristic	No. SARS-CoV-2 IgG positive/no. with data available (%)		Odds ratio (95%CI)	No. SARS-CoV-2 IgG positive/no. with data missing
	True	False		
Individual risk factors				
Pulmonary disease	8/317 (2.5)	99/4,212 (2.4)	1.1 (0.48–2.14)	1/25
Cardiovascular disease	5/329 (1.5)	102/4,200 (2.4)	0.64 (0.22–1.43)	1/25
Diabetes mellitus	5/79 (6.3)	102/4,451 (2.3)	2.96 (1.01–6.81)	1/24
Immunodeficiency	0/92 (0.0)	107/4,434 (2.4)		1/28
Immunosuppressive therapy	1/69 (1.4)	105/4,461 (2.4)	0.7 (0.03–3.15)	2/24
Smoking	11/817 (1.3)	96/3,718 (2.6)	0.52 (0.26–0.94)	1/19
Exposure				
Patient facing role	55/2559 (2.1)	50/1,934 (2.6)	0.83 (0.56–1.22)	3/61
AGPs	9/712 (1.3)	96/3,794 (2.5)	0.50 (0.23–0.94)	3/48
COVID-19 assigned unit	21/712 (2.9)	85/3,803 (2.2)	1.34 (0.80–2.13)	2/39
Emergency department	11/515 (2.1)	95/3,999 (2.4)	0.91 (0.46–1.64)	2/40
Ward	43/1633 (2.6)	63/2,882 (2.2)	1.21 (0.81–1.79)	2/39
Intensive care unit	16/690 (2.3)	89/3,824 (2.3)	1.00 (0.56–1.67)	3/40
Contact with SARS-CoV-2‒positive person				
Patient	31/1028 (3.0)	74/3436 (2.2)	1.42 (0.91–2.15)	3/90
Co-worker	29/816 (3.6)	76/3644 (2.1)	1.74 (1.11–2.65)	3/94
Private contact	22/220 (10.0)	83/4218 (2.0)	5.56 (3.32–8.94)	3/116
Unprotected contact	34/435 (7.8)	70/3997 (1.8)	4.77 (3.09–7.22)	4/122
Protected contact	32/1230 (2.6)	73/3237 (2.3)	1.16 (0.75–1.75)	3/87
Personal protective equipment				
Use of PPE	104/4458 (2.3)	2/75 (2.7)	0.81 (0.25–5.35)	2/21
Surgical mask	104/4437 (2.3)	2/95 (2.1)	1.04 (0.32–6.83)	2/22
FFP2/N95-mask	32/1497 (2.1)	74/3011 (2.5)	0.87 (0.56–1.31)	2/46
FFP3-mask	8/325 (2.5)	96/4163 (2.3)	1.09 (0.48–2.13)	4/66
Protective clothing	34/1677 (2.0)	72/2835 (2.5)	0.8 (0.52–1.19)	2/42
Eye protection or face shield	29/1580 (1.8)	77/2934 (2.6)	0.7 (0.45–1.06)	2/40
Symptoms				
Experienced symptoms	79/1272 (6.2)	28/3263 (0.9)	7.62 (4.98–12.00)	1/19
Exhaustion	54/771 (7.0)	53/3763 (1.4)	5.27 (3.57–7.78)	1/20
Fatigue	67/795 (8.4)	40/3738 (1.1)	8.49 (5.72–12.77)	1/21
Cough	50/668 (7.5)	57/3861 (1.5)	5.40 (3.65–7.97)	1/25
Shortness of breath	19/307 (6.2)	88/4222 (2.1)	3.12 (1.82–5.08)	1/25
Rhinitis	47/689 (6.8)	60/3843 (1.6)	4.62 (3.11–6.82)	1/22
Loss of smell	36/144 (25.0)	71/4384 (1.6)	20.23 (12.87–31.41)	1/26
Loss of taste	39/124 (31.5)	67/4402 (1.5)	29.62 (18.79–46.38)	2/28
Sore throat	30/740 (4.1)	77/3792 (2.0)	2.05 (1.31–3.11)	1/22
Headache	46/766 (6.0)	61/3766 (1.6)	3.88 (2.61–5.73)	1/22
Limb pain	36/403 (8.9)	71/4129 (1.7)	5.61 (3.67–8.45)	1/22
Shivering	36/442 (8.1)	71/4092 (1.7)	5.03 (3.29–7.56)	1/20
Diarrhea	20/316 (6.3)	87/4214 (2.1)	3.22 (1.90–5.21)	1/24
Increased temperature	46/491 (9.4)	61/4032 (1.5)	6.73 (4.51–9.98)	1/31
Fever, temperature >38°C	29/233 (12.4)	77/4288 (1.8)	7.79 (4.90–12.1)	2/33

*AGP, aerosol-generating procedure, COVID-19, coronavirus disease; FFP, filtering face piece; PPE¸ personal protective equipment, SARS-CoV-2, severe acute respiratory syndrome coronavirus 2.

**Table 1:** Seroprevalence of SARS-CoV-2 infections in patients and staff, by general characteristics and occupation, at a university hospital, Munich, Germany*

Characteristic	SARS-CoV-2 IgG, no. (%)		Odds ratio (95% CI)
	Negative	Positive	
Age group, y			
18–30, n = 1,622	1,585 (97.7)	37 (2.3)	Referent
31–40, n = 1,134	1,115 (98.3)	19 (1.7)	0.73 (0.41–1.27)
41–50, n = 758	740 (97.6)	18 (2.4)	1.05 (0.58–1.83)
51–60, n = 766	736 (96.1)	30 (3.9)	1.75 (1.06–2.85)
>60, n = 274	270 (98.5)	4 (1.5)	0.66 (0.19–1.66)
Sex			
F, n = 3,207	3,141 (97.9)	66 (2.1)	Referent
M, n = 1,342	1,300 (96.9)	42 (3.1)	1.54 (1.03–2.27)
Unreported, n = 5	5 (100)	0	
Profession			
Nurses, n = 958	934 (97.5)	24 (2.5)	1.55 (0.80–3.10)
Physicians, n = 860	846 (98.4)	14 (1.6)	Referent
Clinical ancillary staff, n = 383	374 (97.7)	9 (2.3)	1.46 (0.60–3.39)
Nonclinical ancillary staff, n = 120	118 (98.3)	2 (1.7)	1.09 (0.16–4.02)
Scientists/laboratory workers, n = 635	627 (98.7)	8 (1.3)	0.78 (0.31–1.84)
Administrative staff, n = 557	536 (96.2)	21 (3.8)	2.36 (1.19–4.80)
Other, n = 424	412 (97.2)	12 (2.8)	1.76 (0.79–3.88)
Students, n = 603	586 (97.2)	17 (2.8)	1.75 (0.85–3.65)

*SARS-CoV-2, severe acute respiratory syndrome coronavirus 2.

Conversely, we found that seropositivity was particularly high for administrative staff who did not have any direct patient contact (OR 2.36, 95% CI 1.19–4.80) (Table 1; Figure 2, panel B; Appendix Figure 5). Nonclinical staff were not obliged to wear masks at work at the beginning of the pandemic (Figure 1). Seropositivity was also markedly increased in staff who reported exposure to co-workers (OR 1.74, 95% CI 1.11–2.65) or private contacts with persons who had SARS-CoV-2 infections (OR 5.56, 95% CI 3.32–8.94) (Table 2; Figure 2, panel A; Appendix Figure 5). Self-reported unprotected contact with COVID-19 patients (no surgical mask, <1.5-m distance, or AGP without filtering masks with either filtering face piece or N95 standard or eye protection or face shields) was associated with higher seroprevalence (OR 4.77, 95% CI 3.09–7.22) (Table 2; Appendix Figure 5). For staff reporting to perform AGPs we observed an even lower rate of seropositivity (OR 0.50, 95% CI 0.23–0.94) (Table 2; Figure 2, panel A; Appendix Figure 5).

### Symptoms and SARS-CoV-2 IgG Titers for Hospital Staff

In our cohort, 1,272 (27.9%) persons reported current or recent (within 8 weeks before testing) presence of >1 symptom indicative of COVID-19 (Table 2; Appendix Figure 5), 79 (6.2%) of whom were seropositive for SARS-CoV-2 IgG (Table 2; Appendix Figure 5). Loss of smell (36 [25.0%] seropositive of 144 persons who had reported loss of smell) and loss of taste (39 [31.5%] of 124 persons) had the highest positive predictive value (Table 2; Appendix Figure 5), and seropositivity was associated with a higher number of symptoms reported (Table 3; Appendix Figure 5).

**Table 3:** Seroprevalence of SARS-CoV-2 IgG in patients and staff, by symptom onset and frequency, at a university hospital, Munich, Germany*

Characteristic	SARS-CoV-2IgG, no. (%)		Odds ratio (95% CI)
	Negative	Positive	
Symptom onset			
Not applicable, n = 3,373	3,336 (98.9)	37 (1.1)	Referent
Past 14 days, n = 219	209 (95.4)	10 (4.6)	4.36 (2.02–8.59)
Past 3‒8 weeks, n = 943	883 (93.6)	60 (6.4)	6.11 (4.05–9.35)
Unknown, n = 19	18	1	
Symptom frequency, p<0.001			
0, n = 3,273	3,245 (99.1)	28 (0.9)	Referent
1–4, n = 548	529 (96.5)	19 (3.5)	4.17 (2.27–7.50)
5–8, n = 491	454 (92.5)	37 (7.5)	9.42 (5.72–15.70)
9–14, n = 223	200 (89.7)	23 (10.3)	13.32 (7.45–23.58)
Unknown, n = 19	18	1	

*SARS-CoV-2, severe acute respiratory syndrome coronavirus 2.

For seropositive persons, we found no major differences in SARS-CoV-2 IgG titers for different age groups, sex, comorbidities, or exposure profiles (Appendix Figure 6). However, SARS-CoV-2 IgG levels were higher for staff who reported more COVID-19-related symptoms (Appendix Figure 7). We observed the highest titers for those persons who reported diarrhea, fever, increased temperature, shivering, limb pain, and headache (Appendix Figure 7).

### Value of Symptom-Based RT-PCR Testing

We initiated symptom-based RT-PCR testing of material obtained from nasopharyngeal swab specimens early during the pandemic through a dedicated COVID-19 telephone hotline. The first hospital employee with SARS-CoV-2 infection was identified on March 9, 2020, and 28 persons who had SARS-CoV-2 infections detected by RT-PCR before participating in this study were positive for SARS-CoV-2 IgG (Figure 1; Appendix Figure 8). Ten seropositive persons had a positive PCR result; 1 positive antibody testing result was obtained at another facility. However, 68 (63%) of 108 SARS-CoV-2 infections had not been diagnosed previously; data on previous testing was missing for 1 person. Among these 68 seropositive persons, 28 did not report any COVID-19-typical symptoms in the initial survey (25.9% of all seropositive staff), indicating that symptom-based testing can miss SARS-CoV-2 infection.

### Analysis of Spatiotemporal Trajectories

**Figure 3:**
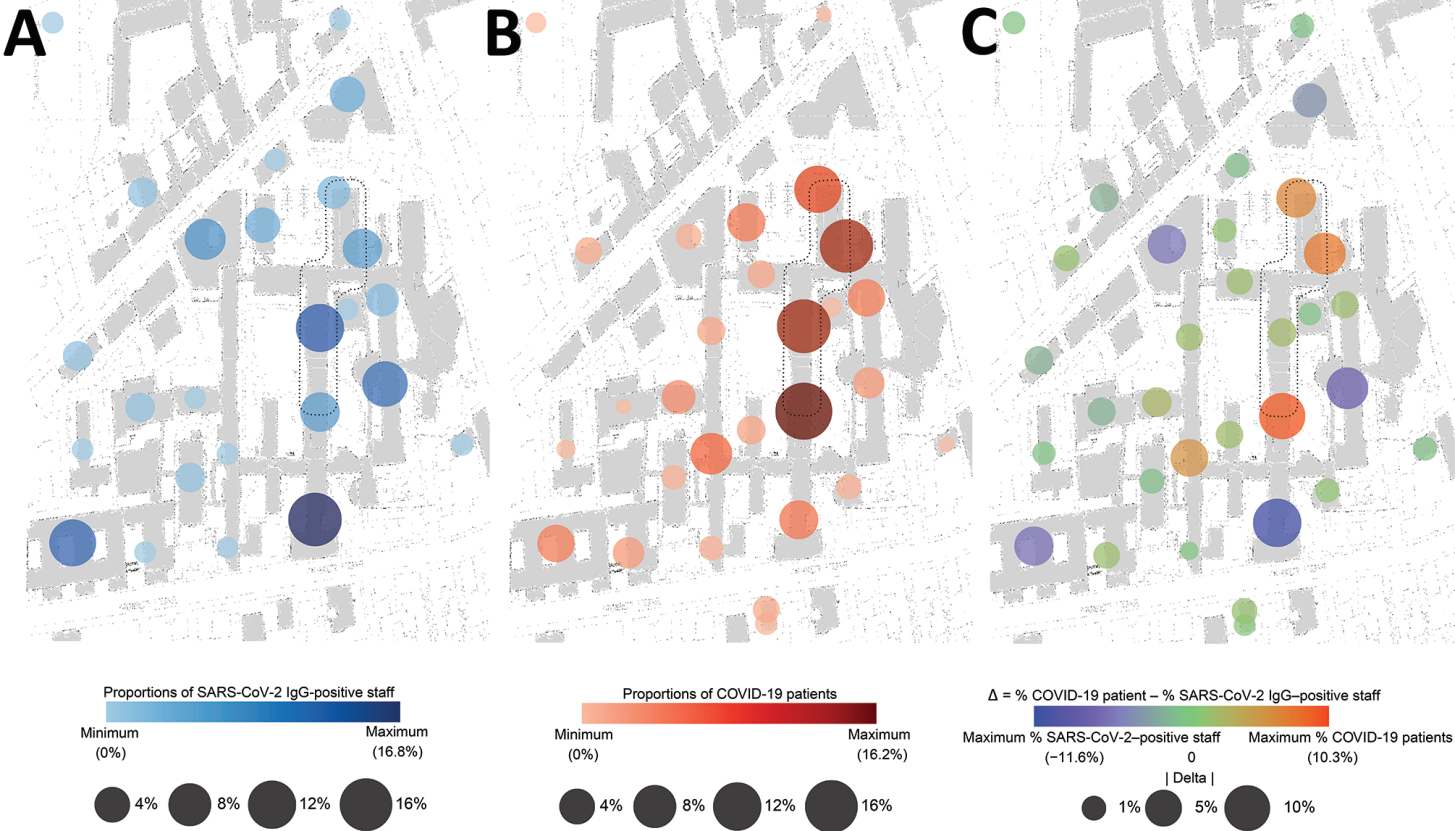
Spatiotemporal trajectories of severe acute respiratory syndrome coronavirus 2‒infected patients and staff mobility in university hospital, Munich, Germany. A) Cumulative representation of proportions of seropositive staff. B) Cumulative representation of proportions of COVID-19-patients. C) Differences (Δ) for staff and patients between different hospital areas. Difference are indicated by dot plots and assigned to distinct hospital areas. For purposes of discretion of data from study participants, the graphic representation of spatial information is partially distorted. Dashed lines indicate COVID-19‒designated areas in the hospital.

To identify and localize potential hotspots of infection, we systematically analyzed contact between staff and COVID-19 patients by using the cumulative data for serologic analysis for staff and the patient registry. Thus, we plotted available spatial and temporal information on the presence of COVID-19 patients and SARS-CoV-2 IgG‒positive staff with daily resolution on a hospital map. Visualization of these spatiotemporal mobility trajectories showed only a slight overlap between the distinct spatial and temporal hotspots of COVID-19 patients and SARS-CoV-2 IgG‒positive staff (Figure 3; Videos 1–3).

**Video 1:** Time lapse of relative proportions of all trajectories for coronavirus disease (COVID-19) cases. Proportions in distinct hospital locations were normalized by all timeframes during February 1‒May 29, 2020. Some patients who later tested positive for severe acute respiratory syndrome coronavirus 2 were already in the hospital before March 6, 2020, and are therefore visualized starting February 1.

**Video 3:** Time lapse of relative proportions for the difference in trajectories available for coronavirus disease (COVID-19) cases and for severe acute respiratory syndrome coronavirus 2‒positive staff. Proportions in distinct hospital locations were normalized by all timeframes during February 1‒May 29, 2020. For purposes of discretion, graphic representation of spatial information is partially distorted.

## Discussion

Despite the high overall number of patients in our hospital who had COVID-19 disease, the seroprevalence of 2.4% for SARS-CoV-2 IgG among university hospital staff after the first wave in Germany is lower than that reported in previous studies (*11**,**12*). This difference might be attributed to differences in cohort composition, fast implementation of protective measures, or frequency of exposure.

Hospital staff have an increased occupational risk for contact with SARS-CoV-2‒infected patients, and a high level of SARS-CoV-2 infection among HCWs involved in the care of COVID-19 patients has been reported (*7*,*13*). Consistent with these findings, the seropositivity in a population-based prospective cohort study performed in Munich in parallel with our study was 1.8% and thus lower than for this HCW cohort study (*14*). We did not observe higher seroprevalences in staff who reported direct patient contact, including those working in COVID-19‒designated units. We also observed lower seroprevalence in staff who reported performing AGPs, possibly reflecting increased awareness and use of particularly rigorous infection prevention practices at work and in private life in this subgroup. Furthermore, the type of PPE used was not associated with seroprevalence, but 98% of staff reported routinely using surgical masks, which was required by internal hospital policy for staff involved in patient care starting on March 16, 2020, and for all staff starting on March 27. The almost parallel increase in SARS-CoV-2 infection cases in staff and patients is suggestive of extrinsic infection causes in both groups, such as simultaneous return from high-risk holiday areas. Consistently, exposure to SARS-CoV-2-infected private contacts or co-workers was the most critical risk factor for SARS-CoV-2 infection in our cohort. This finding underscores the need for adherence to protective measures during private, professional staff, and professional patient contacts.

Male staff in our study cohort showed a higher seroprevalence. A recent study reporting lower perceived infection risk for men than for women found that adherence to hygiene guidelines and social distancing measures might have been lower in male staff (*15*,*16*). Smokers showed a lower seroprevalence, which is in contrast to that reported in previous studies (*2*,*6*,*15*,*17*). Because smokers are more susceptible to respiratory tract infections and smoking involves hand-to-mouth contact and frequent social interactions, the lower seroprevalence is unexpected but consistent with those of other reports (*18**–**21*). Staff who had diabetes mellitus had a higher seroprevalence than staff who did not have this disease. Previously, diabetes mellitus reportedly correlated with severity of COVID-19 and associated deaths, but no increase in susceptibility to SARS-CoV-2 infection has been reported to date (*22*).

Serologic assessment confirmed infection in most persons who reported positive test results for SARS-CoV-2 by PCR. However, repeated testing over >4 weeks with >2 separate assays each did not detect antibodies in 6 persons (2 who had positive in-house and 4 who had reported positive external RT-PCR test results) (Appendix Figure 8). This finding might be explained by false-positive PCR results or by the failure to develop antibody responses after low-symptom infection, which might occur in <10% of convalescent-phase patients after SARS-CoV-2 infection (*23*).

The IgG immunoassay used for screening had a specificity of 99.89% in our study; it uses 2 SARS-CoV-2 antigens (nucleocapsid and spike 1) for detection and has an estimated sensitivity of 96.30% (Appendix Table 5). We retested all IgM-positive or IgG-positive serum samples and all serum samples that had titers of 5–10 AU/mL, which is below the cutoff, by a second assay with particularly high specificity (99.90%) that uses recombinant nucleocapsid protein as antigen. If required, additional assays were performed: either an ELISA using recombinant spike 1 protein as capture antigen, or immunoblotting that tested for antibodies against 3 different SARS-CoV-2 antigens. Serum was only considered positive if >2 antibody assay results were positive (Appendix). However, the requirement of such extensive confirmatory testing strengthens the notion that each test for SARS-CoV-2 antibodies requires critical evaluation.

Our study also showed that approximately two thirds of the seropositive staff had a previously undetected infection. These infections might have been oligosymptomatic or asymptomatic without even alarming medically trained personnel. Furthermore, 25 SARS-CoV-2 IgG‒positive persons had not been PCR tested, despite reporting >1 COVID-19‒compatible symptom. A total of 1,183 staff members tested seronegative despite reporting >1 symptom related to COVID-19. The focus on symptoms with the strongest association with seropositivity, such as loss of smell, loss of taste, fatigue, fever, and cough, might therefore be helpful in developing more accurate and economical screening algorithms. Our results highlight that symptom-based testing might miss infections in hospital staff. All 28 asymptomatic seropositive persons remained undiagnosed before the study, emphasizing the need for rigorous implementation of systematic infection prevention practices in pandemic situations. Transmission by asymptomatic and presymptomatic staff might occur at any time and will not be prevented by random testing. These results strongly support the continuous use of at least surgical masks as a simple and efficient measure for employee and patient protection.

To identify infection hotspots and putative patient/staff overlaps, we visualized the temporospatial mobility trajectories of patients and staff to monitor the infection dynamics. Real-time use of such trajectory mapping at high resolution might yield additional information that enables the reaction to procced more quickly and intuitively to infection foci. Continuous evaluation of mobility trajectory mappings might highlight areas of recurrent infections and thus identify previously unattended needs that should be addressed for future waves of the pandemic.

Our study’s first limitation is that because this was a voluntary assessment, participation was incomplete and might have biased the results. We cannot exclude the possibility that staff members with a higher perceived risk for infection were more likely to participate. Second, symptoms and exposures were retrospectively assessed and self-reported and thus subject to a recall bias in participants knowing of their SARS-CoV-2 infection. Third, we did not assess individual adherence of mask wearing in our questionnaire, especially regarding specific, potentially hazardous situations (e.g., during breaks, in locker rooms). Consequently, this approach does not enable us to pinpoint risk for work-related infection to specific situations.

In addition, because it was not obligatory to indicate in our questionnaire the periods during which masks were worn, the analysis reflects the protective effect of masks over the entire period. Furthermore, although we attempted a cross-sectional analysis, our data document average seroprevalence during the entire testing period. Thus, seroconversions occurring during this period might have been missed. Finally, RT-PCR testing results were available only for persons who consented to their use (4,373/4,554), limiting the possibility of cross-validating PCR-testing results with seroprevalence.

Our findings have several major implications. The infection rate for HCWs was not markedly increased, and infections occurred in parallel to the general population. We did not observe a relevant increase in SARS-CoV-2 IgG seropositivity in HCWs (including those working with COVID-19 patients) compared with staff who were not directly involved in patient care, as long as PPE was used, suggesting that PPE and other infection control practices successfully prevented transmission from SARS-CoV-2‒infected patients. Interaction with SARS-CoV-2‒infected co-workers or private contacts was a major risk factor for infection. The infection rate among HCWs seemed to decrease when wearing surgical face masks became obligatory in all areas of the hospital. Thus, obligatory wearing of certified surgical masks by all employees, no matter when in contact with patients, relatives, or colleagues, and, whenever tolerated, also by patients, might minimize virus transmission risks. However, it was not possible to formally separate that effect from that of minimizing personal contacts imposed by the general lock-down and a concomitant decrease in COVID-19 incidence.

In summary, the value of SARS-CoV-2 antibodies for protective immunity and their sustainability in infected persons remains unclear. Longitudinal studies with combined testing for virus-specific antibodies and their infection-neutralizing ability, as well as virus-specific T-cell immunity, are needed to estimate the longevity and protective value of SARS-CoV-2 IgG responses in hospital staff. However, our results show that patient-facing healthcare work during the SARS-CoV-2 pandemic might be safe as long as adequate PPE is used and infection prevention practices are followed, both inside and outside the hospital.

**Video 2:** Time lapse of relative proportions of trajectories available for severe acute respiratory syndrome coronavirus 2‒positive staff working on campus Proportions were mapped for 14 days on their last location before staff either tested positive or were sent to quarantine. Results were normalized by all timeframes during March 22‒May 29, 2020.

Appendix Additional information on Infection control measures and prevalence of severe acute respiratory syndrome coronavirus 2 IgG among 4,554 university hospital employees, Munich, Germany.
