# Supplementary figures and images for: Infection Control Measures and Prevalence of SARS-CoV-2 IgG among 4,554 University Hospital Employees, Munich, Germany
Source: Emerg Infect Dis. 2022 Mar;28(3):572–81. doi: 10.3201/eid2803.204436 (PMC8888242; doi:10.3201/eid2803.204436)

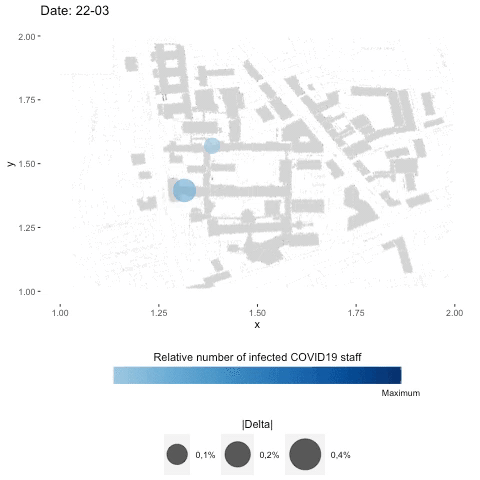

Supplement: Supplementary file 1 [file 20-4436-V1.gif]

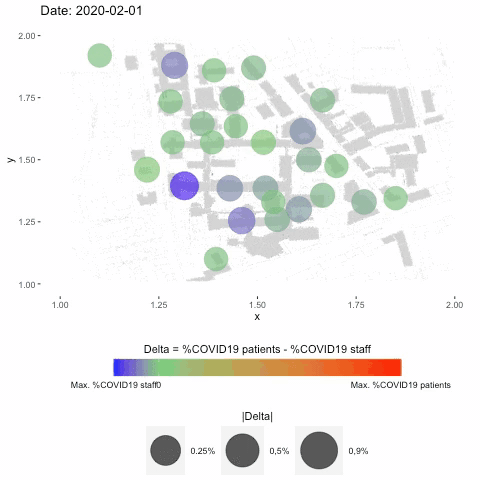

Supplement: Supplementary file 2 [file 20-4436-V3.gif]
