# Supplementary material for: Infection Control Measures and Prevalence of SARS-CoV-2 IgG among 4,554 University Hospital Employees, Munich, Germany
Source: Emerg Infect Dis. 2022 Mar;28(3):572–81. doi: 10.3201/eid2803.204436 (PMC8888242; doi:10.3201/eid2803.204436)
Supplement: Appendix — Additional information on Infection control measures and prevalence of severe acute respiratory syndrome coronavirus 2 IgG among 4,554 university hospital employees, Munich, Germany. [file 20-4436-Techapp-s1.pdf]

Article DOI: https://doi.org/10.3201/eid2803.204436

Infection Control Measures and Prevalence of SARS-CoV-2 IgG among 4,554 University Hospital Employees, Munich, Germany

Appendix

Questionnaire

The original survey was in German and was acquired by using a standardized, electronic questionnaire.

Baseline characteristics

Date of assessment ☐☐.☐☐.☐☐☐☐

Baseline characteristic: Age, sex

Department:

Worksite:

Occupation

☐ Physician ☐ Nurse

☐ Lab Worker ☐ Hygiene Staff ☐ Clinical Ancillary Staff ☐ Cleaning Staff ☐ Patient Transport

☐ Administration ☐ Technical Staff ☐ IT ☐ Scientist ☐Student ☐ Others

Do you have a patient facing role? ☐ Yes ☐ No

Exposure and personal protective equipment

In which area(s) have you been placed? (multiple answers possible)

Work area	Currently	Past 48 hours	Past 3 to 14 days	Past 3 to 8 weeks
COVID-19 assigned area				
Emergency department				
Ward				
Intensive care unit				
Other				
Aerosol generating procedures*				

*Endoscopy, bronchoscopy, tracheal intubation, non-invasive ventilation, transesophageal echo, etc.

Have you been in contact with SARS-CoV-2-positive individuals? ☐ Yes ☐ No

COVID-19 contact	Currently	Past 48 hours	Past 3 to 14 days	Past 3 to 8 weeks
Patients at MRI				
Co-worker at MRI				
Private contact				
Protected (mask and physical distance, or FFP2/N95 and eye protection when performing aerosol-generating procedures				
Unprotected (none of the abovementioned, or mask only when performing aerosol generating procedures				

Do you use personal protective equipment? ☐ Yes ☐ No

If so, which ones? (multiple answers possible)

Personal protective equipment	Currently	Past 48 hours	Past 3 to 14 days	Past 3 to 8 weeks
Mask				
FFP2/N95				
FFP3				
Protective clothing				
Eye protection or face shield				
Others				

Individual factors

What applies to you (multiple answers possible)?

☐ Smoking ☐ Pulmonary disease ☐ Cardiovascular disease ☐ Diabetes mellitus

☐ Immunodeficiency ☐ Immunosuppressive therapy

☐ Other

Have you had COVID-19 compatible symptoms? ☐ Yes ☐ No (multiple answers possible)

Symptoms	Currently	Past 48 hours	Past 3 to 14 days	Past 3 to 8 weeks
Exhaustion				
Fatigue				
Cough				
Shortness of breath				
Rhinitis				
Loss of smell				
Loss of taste				
Sore throat				
Headache				
Limb pain				

Shivering				
Diarrhea				
Elevated temperature (37.3–37.9°C)				
Fever (>38°C)				
Current body temperature ☐☐ . ☐☐ °C				

Have you ever been tested for SARS-CoV-2? ☐ Yes ☐ No

if so: ☐ Past 14 days ☐ More than 14 days ago

Where? ☐ MRI ☐ Registered physician ☐ Department Of Public Order

How? ☐ Nasopharyngeal swab ☐ Blood ☐ Stool

Test result:

☐ Pending ☐ Positive for SARS-COV-2 ☐ Negative for SARS-CoV-2

COVID-19 disease

Have you already had COVID-19? ☐ Yes ☐ No

Treatment: ☐ Outpatient/at home ☐ Inpatient/normal ward ☐ Ward and intensive care unit

Calculation of Specificity and Sensitivity of the SARS-CoV-2 Antibody Tests

IgG and IgM were determined in 4,554 and 1,708 serum samples, respectively, by using a paramagnetic particle chemiluminescent immunoassay (CLIA) on an iFlash 1800 immunoassay analyzer (Shenzhen Yhlo Biotech Co., Shenzhen, China). This assay was selected as a screening assay because it detects antibodies directed against either SARS-CoV-2 S1 or N protein. According to the manufacturer’s instructions, values ≥10 AU/mL were considered positive. SARS-CoV-2 IgG titers were positive (≥10 AU/L) in 108 persons, negative (<5 AU/L) in 4,411 persons, and 35 persons had borderline results (5–10 AU/mL) (Appendix Figure 5).

To determine the sensitivity and specificity of the screening assay, confirmatory testing was performed in all serum samples that tested positive for IgM or IgG, all serum samples with

IgG values between 5 and 10 AU/mL, and all serum samples from SARS-CoV-2 PCR-positive persons. For confirmation, the total antibodies against SARS-CoV-2 N protein were determined by using an electrochemiluminescent immunoassay (ECLIA) on a Cobas e411 analyzer (Roche Diagnostics, Mannheim, Germany). In all samples with incongruent results, IgG against SARS-CoV-2 S1 protein were determined by using an ELISA (Euroimmun, Luebeck, Germany), while immunoblot was used to differentiate antibodies against N, S1, and the receptor binding domain (RBD) of SARS-CoV-2 from those against seasonal coronaviruses (Mikrogen, Neuried, Germany).

Tests were considered correct if the presence of antibodies was confirmed by at least one more independent assay. Of the 108 serum samples that tested positive in the Yhlo screening assay, 93 also tested positive in the Roche IgG assay, eight were confirmed by immunoblotting (Appendix Tables 1 and 4). In one individual the screening result was considered specific due to high IgG titer and concomitant IgM positivity although no confirmatory testing could be performed (Appendix Tables 1 and 4, Sample-ID 18). In another individual testing only IgG positive, the serum amount was insufficient for confirmatory testing (Appendix Table 3, Sample-ID 114). Five IgG test results were considered false positive because screening IgG results were not confirmed by any of the other assays (Appendix Table 3). This resulted in a specificity of 99.89% for the IgG assay (4,441/4,446; Appendix Table 5).

IgM was screened in all patients until May 4 (n = 1,620). Six patients lacking prevalence of SARS-CoV-2 IgG tested positive for IgM (6/1,620). Because this could not be confirmed by the Roche ECLIA detecting IgM and IgG, these samples were considered false positive (S4 Table). If the Roche assay would have a 100% sensitivity, this would result in a specificity of 99.63% for the IgM assay. Due to the lack of a third assay, this, however, has to be considered preliminary.

To determine the sensitivity of the Yhlo IgG screening assay, 35 samples with detectable values between 5 and 10 AU/mL, i.e., below the recommended cutoff of the assay, were retested with both the Roche and the Euroimmun assay. Four samples tested positive in the Roche and Euroimmun assays, and were therefore considered false negative in the Yhlo screening assay (Appendix Tables 1 and 4). This enabled us to estimate the overall sensitivity of the IgG assay at 96.30% (104/108; Appendix Table 5).

For estimation of seroprevalence, persons who had ≥2 positive antibody test results (n = 106) as well as 2 persons with positive SARS-CoV-2 PCR tests that seroconverted during follow-up, were considered seropositive (108/4,554) resulting in a seroprevalence of 2%–4%. The IgG levels of seropositive persons were inversely correlated with the time of testing (rho = −0.22, [95% CI −0.39 to −0.03]) (Appendix Figure 5).

From May 5, persons were tested for SARS-CoV-2 IgM if specific SARS-CoV-2 IgG was detected or typical symptoms of COVID-19 were reported (n = 88). Overall, concomitant SARS-CoV-2 IgG and IgM was found in 22 patients (22/1,708), of these nine before May 5 (Appendix Figure 2).

Appendix Table 1. Samples with confirmed positive IgG against SARS-CoV-2

Sample ID	SARS-CoV-2 PCR	YHLO IgG (AU/mL)	YHLO IgM (AU/mL)	Roche IgG ‡ IgM (COI)	Euroimmun IgG	Mikrogen recomLine
1	••	POSITIVE (80.12)	POSITIVE (19.27)	POSITIVE (14.42)	••	••
2	••	POSITIVE (92.39)	POSITIVE (17.93)	POSITIVE (52.30)	••	••
3	••	POSITIVE (64.82)	POSITIVE (31.04)	POSITIVE (25.83)	••	••
4	§	POSITIVE (113.83)	NEGATIVE (1.78)	POSITIVE (66.12)	••	••
5	§‡	POSITIVE (93.54)	NEGATIVE (2.43)	POSITIVE (21.33)	••	••
6	§	POSITIVE (109.62)	NEGATIVE (3.46)	POSITIVE (49.23)	••	••
7	••	POSITIVE (102.26)	NEGATIVE (2.36)	POSITIVE (17.38)	••	••
8	••	POSITIVE (33.60)	NEGATIVE (2.80)	POSITIVE (9.08)	••	••
9	••	POSITIVE (28.28)	NEGATIVE (2.80)	NEGATIVE (0.06)	NEGATIVE	POSITIVE
10	••	POSITIVE (38.15)	NEGATIVE (4.40)	POSITIVE (13.05)	••	••
11	••	POSITIVE (113.58)	NEGATIVE (3.05)	POSITIVE (65.42)	••	••
12	§	POSITIVE (96.21)	NEGATIVE (1.91)	POSITIVE (47.08)	••	••
13	§	POSITIVE (78.86)	NEGATIVE (2.29)	POSITIVE (32.81)	••	••
14	‡	POSITIVE (107.45)	NEGATIVE (0.59)	POSITIVE (14.49)	••	••
15	••	POSITIVE (41.99)	NEGATIVE (2.59)	POSITIVE (2.98)	••	••
16	‡	POSITIVE (69.45)	NEGATIVE (0.60)	NEGATIVE (0.055)	NEGATIVE	POSITIVE
17	••	POSITIVE (84.41)	NEGATIVE (5.78)	POSITIVE (40.43)	••	••
18	••	POSITIVE (78.97)	POSITIVE (34.76)	* ••	*	••
19	§‡	POSITIVE (12.45)	NEGATIVE (0.83)	NEGATIVE (0.45)	BORDERLINE	POSITIVE
20	••	POSITIVE (86.18)	NEGATIVE (1.14)	POSITIVE (19.89)	••	••
21	••	POSITIVE (22.12)	NEGATIVE (0.73)	POSITIVE (1.81)	••	••
22	••	POSITIVE (92.35)	POSITIVE (19.96)	POSITIVE (28.59)	••	••
23	••	POSITIVE (100.84)	POSITIVE (11.19)	POSITIVE (84.33)	••	••
24	••	POSITIVE (30.86)	NEGATIVE (0.48)	NEGATIVE (0.054)	NEGATIVE	POSITIVE
25	‡	POSITIVE (95.56)	NEGATIVE (6.06)	POSITIVE (27.94)	••	••
26	••	POSITIVE (91.06)	NEGATIVE (1.41)	POSITIVE (48.79)	••	••
27	••	POSITIVE (25.61)	NEGATIVE (1.89)	POSITIVE (4.55)	••	••
28	§	POSITIVE (35.34)	NEGATIVE (1.67)	POSITIVE (7.32)	••	••
29	••	POSITIVE (72.10)	POSITIVE (22.39)	POSITIVE (75.58)	••	••
30	§	POSITIVE (97.45)	NEGATIVE (4.91)	POSITIVE (65.48)	••	••
31	§	POSITIVE (59.51)	POSITIVE (10.26)	POSITIVE (79.13)	••	••
32	••	POSITIVE (42.14)	POSITIVE (17.68)	POSITIVE (14.92)	••	••
33	§	POSITIVE (49.91)	NEGATIVE (1.19)	POSITIVE (27.04)	••	••
34	§‡	POSITIVE (97.02)	NEGATIVE (10.00)	POSITIVE (99.14)	••	••
35	§‡	POSITIVE (91.68)	NEGATIVE (1.89)	POSITIVE (14.13)	••	••
36	••	POSITIVE (35.20)	NEGATIVE (1.98)	POSITIVE (2.63)	••	••
37	§	POSITIVE (55.52)	NEGATIVE (0.53)	POSITIVE (10.45)	••	••
38	••	POSITIVE (27.81)	NEGATIVE (0.48)	POSITIVE (14.44)	••	••
39	••	POSITIVE (98.57)	NEGATIVE (4.07)	POSITIVE (70.04)	••	••
40	••	POSITIVE (86.47)	NEGATIVE (9.97)	POSITIVE (89.22)	••	••
41	••	POSITIVE (12.51)	NEGATIVE (1.06)	POSITIVE (2.87)	••	••
Sample ID	SARS-CoV-2 PCR	YHLO IgG (AU/mL)	YHLO IgM (AU/mL)	Roche IgG ‡ IgM (COI)	Euroimmun IgG	Mikrogen recomLine
42	••	POSITIVE (45.40)	NEGATIVE (0.79)	POSITIVE (21.43)	••	••
43	§‡	POSITIVE (40.13)	NEGATIVE (1.65)	POSITIVE (25.15)	••	••
44	§‡	POSITIVE (35.65)	NEGATIVE (9.91)	POSITIVE (51.67)	••	••

Sample ID	SARS-CoV-2 PCR	YHLO IgG (AU/mL)		YHLO IgM (AU/mL)		Roche IgG ‡ IgM (COI)		Euroimmun IgG	Mikrogen recomLine
45	§‡	POSITIVE	(91.08)	NEGATIVE	(0.43)	POSITIVE	(89.17)	••	••
46	••	POSITIVE	(45.18)	NEGATIVE	(1.76)	POSITIVE	(30.29)	••	••
47	§‡	POSITIVE	(113.23)	NEGATIVE	(2.61)	POSITIVE	(40.58)	••	••
48	••	POSITIVE	(73.79)	NEGATIVE	(0.67)	POSITIVE	(43.39)	••	••
49	••	POSITIVE	(93.50)	POSITIVE	(10.14)	POSITIVE	(27.46)	••	••
50	§‡	POSITIVE	(84.28)	NEGATIVE	(0.68)	POSITIVE	(50.96)	••	••
51	§‡	POSITIVE	(96.75)	NEGATIVE	(4.80)	POSITIVE	(100.30)	••	••
52	••	POSITIVE	(36.27)	NEGATIVE	(0.85)	POSITIVE	(2.72)	••	••
53	••	POSITIVE	(10.52)	NEGATIVE	(0.37)	NEGATIVE	(0.061)	NEGATIVE	POSITIVE
54	§	POSITIVE	(49.49)	NEGATIVE	(0.94)	POSITIVE	(39.16)	••	••
55	••	POSITIVE	(50.98)	NEGATIVE	(1.54)	POSITIVE	(78.15)	••	••
56	§‡	POSITIVE	(83.97)	NEGATIVE	(5.40)	POSITIVE	(45.04)	••	••
57	‡	POSITIVE	(32.83)	NEGATIVE	(0.24)	POSITIVE	(19.70)	••	••
58	§	POSITIVE	(72.67)	NEGATIVE	(3.55)	POSITIVE	(11.77)	••	••
59	••	POSITIVE	(60.13)	NEGATIVE	(2.74)	NEGATIVE	(0.055)	NEGATIVE	POSITIVE
60	••	POSITIVE	(66.95)	NEGATIVE	(2.27)	POSITIVE	(39.01)	••	••
61	‡	POSITIVE	(47.80)	NEGATIVE	(1.08)	POSITIVE	(19.42)	••	••
62	••	POSITIVE	(82.89)	NEGATIVE	(4.19)	POSITIVE	(60.83)	••	••
63	§	POSITIVE	(23.44)	NEGATIVE	(0.42)	POSITIVE	(14.57)	••	••
64	••	POSITIVE	(20.75)	NEGATIVE	(1.49)	POSITIVE	(21.14)	••	••
65	••	POSITIVE	(45.24)	NEGATIVE	(0.72)	POSITIVE	(53.24)	••	••
66	••	POSITIVE	(33.84)	NEGATIVE	(0.29)	POSITIVE	(17.67)	••	••
67	••	POSITIVE	(14.69)	NEGATIVE	(0.49)	POSITIVE	(10.88)	••	••
68	‡	POSITIVE	(54.55)	POSITIVE	(22.09)	POSITIVE	(62.24)	••	••
69	‡	POSITIVE	(46.11)	NEGATIVE	(0.95)	POSITIVE	(36.16)	••	••
70	‡	POSITIVE	(10.01)	NEGATIVE	(1.34)	POSITIVE	(9.90)	••	••
71	••	POSITIVE	(52.17)	NEGATIVE	(0.62)	POSITIVE	(32.00)	••	••
72	••	POSITIVE	(13.79)	NEGATIVE	(0.73)	POSITIVE	(8.11)	••	••
73	••	POSITIVE	(69.38)	POSITIVE	(29.99)	POSITIVE	(91.78)	••	••
74	••	POSITIVE	(68.40)	NEGATIVE	(4.42)	POSITIVE	(90.23)	••	••
75	••	POSITIVE	(27.70)	NEGATIVE	(0.40)	POSITIVE	(6.11)	••	••
76	••	POSITIVE	(82.56)	POSITIVE	(19.32)	POSITIVE	(102.00)	••	••
77	••	POSITIVE	(58.50)	NEGATIVE	(0.41)	POSITIVE	(84.81)	••	••
78	§‡	POSITIVE	(74.31)	NEGATIVE	(0.78)	POSITIVE	(68.42)	••	••
79	••	POSITIVE	(69.18)	NEGATIVE	(0.50)	POSITIVE	(81.05)	••	••
80	••	POSITIVE	(35.63)	NEGATIVE	(1.11)	POSITIVE	(62.51)	••	••
81	‡	POSITIVE	(64.48)	NEGATIVE	(0.80)	POSITIVE	(56.41)	••	••
82	••	POSITIVE	(82.53)	POSITIVE	(22.54)	POSITIVE	(81.01)	••	••
83	••	POSITIVE	(10.52)	NEGATIVE	(0.48)	NEGATIVE	(0.053)	NEGATIVE	POSITIVE
Sample ID	SARS-CoV-2 PCR	YHLO IgG (AU/mL)		YHLO IgM (AU/mL)		Roche IgG ‡ IgM (COI)		Euroimmun IgG	Mikrogen recomLine
84	••	POSITIVE	(68.97)	NEGATIVE	(2.00)	POSITIVE	(51.00)	••	••
85	••	POSITIVE	(80.39)	NEGATIVE	(5.54)	POSITIVE	(94.25)	••	••
86	‡	POSITIVE	(52.70)	POSITIVE	(30.33)	POSITIVE	(40.29)	••	••
87	••	POSITIVE	(35.43)	NEGATIVE	(0.36)	POSITIVE	(16.54)	••	••
88	§	POSITIVE	(27.04)	NEGATIVE	(5.83)	POSITIVE	(1.15)	••	••
89	••	POSITIVE	(75.74)	NEGATIVE	(2.60)	POSITIVE	(85.36)	••	••
90	••	POSITIVE	(57.12)	NEGATIVE	(2.73)	NEGATIVE	(0.055)	NEGATIVE	POSITIVE
91	••	POSITIVE	(26.50)	NEGATIVE	(0.45)	POSITIVE	(52.46)	••	••
92	••	POSITIVE	(63.67)	NEGATIVE	(0.46)	POSITIVE	(31.93)	••	••
93	••	POSITIVE	(56.33)	NEGATIVE	(0.67)	POSITIVE	(100.70)	••	••
94	••	POSITIVE	(85.75)	NEGATIVE	(0.66)	POSITIVE	(112.80)	••	••
95	••	POSITIVE	(41.98)	NEGATIVE	(0.63)	POSITIVE	(87.69)	••	••
96	••	POSITIVE	(14.15)	NEGATIVE	(0.97)	POSITIVE	(11.00)	••	••
97	§	POSITIVE	(83.09)	NEGATIVE	(1.92)	POSITIVE	(112.00)	••	••
98	••	POSITIVE	(43.75)	NEGATIVE	(0.63)	POSITIVE	(77.74)	••	••
99	‡	POSITIVE	(54.46)	NEGATIVE	(22.17)	POSITIVE	(73.22)	••	••
100	§‡	POSITIVE	(86.86)	NEGATIVE	(1.45)	POSITIVE	(70.92)	••	••
101	••	POSITIVE	(15.87)	NEGATIVE	(1.08)	POSITIVE	(3.05)	••	••
102	§‡	POSITIVE	(63.04)	NEGATIVE	(5.02)	POSITIVE	(120.80)	••	••
103	§‡	NEGATIVE	(6.55)	••	••	POSITIVE	(1.76)	POSITIVE	••
104	‡	NEGATIVE	(5.26)	••	••	POSITIVE	(2.22)	POSITIVE	••
105	••	NEGATIVE	(8.81)	••	••	POSITIVE	(5.85)	POSITIVE	••
106	‡	NEGATIVE	(6.64)	••	••	POSITIVE	(1.75)	POSITIVE	••
107†	§	POSITIVE†	(37.99)	POSITIVE †	(20.47)	••	••	••	••
108†	§	POSITIVE†	(45.70)	NEGATIVE	(1.14)	••	••	••	••

*No material for further tests available, † positive at follow-up visit, COI: Cutoff index, ‡ positive SARS-CoV-2 PCR extern, § positive SARS-CoV-2 PCR in-house, •• not available.

Appendix Table 2. Summary of confirmatory assays

YHLO IgG (AU/mL)	YHLO IgM (AU/mL)	Roche IgG‡ IgM (COI)	Euroimmun IgG (ratio)	Mikrogen recomLine immunoblot	Final result	No. patients
POSITIVE	••	POSITIVE	••	••	POSITIVE	93
POSITIVE	••	NEGATIVE	NEGATIVE	POSITIVE	POSITIVE	8
POSITIVE	NEGATIVE	NEGATIVE	NEGATIVE/BORDERLINE	NEGATIVE	NEGATIVE	5
POSITIVE	POSITIVE	*	*	*	POSITIVE	1
POSITIVE	NEGATIVE	*	*	NEGATIVE	Excluded from calculation of specificity	1
BORDERLINE	••	POSITIVE	POSITIVE	••	POSITIVE	4
POSITIVE†	••	••	••	••	POSITIVE	2[§]

†Initially negative, but positive at follow-up visit, * no material for further tests available, [§] in-house SARS-CoV-2 PCR positive, COI: Cutoff index.

Appendix Table 3. Samples with positive IgG against SARS-CoV-2 that could not be confirmed

Sample ID	YHLO IgG (AU/mL)		YHLO IgM (AU/mL)		Roche IgG ‡ IgM (COI)		Euroimmun IgG	Mikrogen recomLine
109	POSITIVE	(26.78)	NEGATIVE	(0.19)	NEGATIVE	(0.079)	NEGATIVE	NEGATIVE
110	POSITIVE	(23.75)	NEGATIVE	(1.17)	NEGATIVE	(0.055)	BORDERLINE	NEGATIVE
111	POSITIVE	(10.14)	NEGATIVE	(0.82)	NEGATIVE	(0.126)	NEGATIVE	NEGATIVE
112	POSITIVE	(21.46)	NEGATIVE	(0.44)	NEGATIVE	(0.102)	NEGATIVE	NEGATIVE
113	POSITIVE	(12.13)	NEGATIVE	(0.50)	NEGATIVE	(0.055)	NEGATIVE	NEGATIVE
114	POSITIVE	(11.75)	NEGATIVE	(0.28)	*	••	*	••

*No material for further tests available.

Appendix Table 4. Samples with positive IgM against SARS-CoV-2 that could not be confirmed

Sample ID	YHLO IgG (AU/mL)		YHLO IgM (AU/mL)		Roche IgG ‡ IgM (COI)	
115	NEGATIVE	(0.98)	POSITIVE	(11.51)	NEGATIVE	(0.054)
116	NEGATIVE	(0.41)	POSITIVE	(13.71)	NEGATIVE	(0.055)
117	NEGATIVE	(2.90)	POSITIVE	(13.41)	NEGATIVE	(0.055)
118	NEGATIVE	(0.28)	POSITIVE	(12.23)	NEGATIVE	(0.056)
119	NEGATIVE	(0.45)	POSITIVE	(10.27)	NEGATIVE	(0.056)
120	NEGATIVE	(0.14)	POSITIVE	(265.82)	NEGATIVE	(0.056)

Appendix Table 5. Calculation of specificity and sensitivity

Result	≥2 Positive confirmatory tests or concomitant IgM	<2 Positive confirmatory tests	Total
Screening assay positive	True positive, n = 104	False positive, n = 5	n = 109†
Screening assay negative	False negative, n = 4	True negative, n = 4,441	n = 4,445
Total	n = 108	n = 4,446	4,554

†Serum material was insufficient for confirmatory testing for 1 seropositive patients; 2 patients showed seroconversion at follow-up visit.

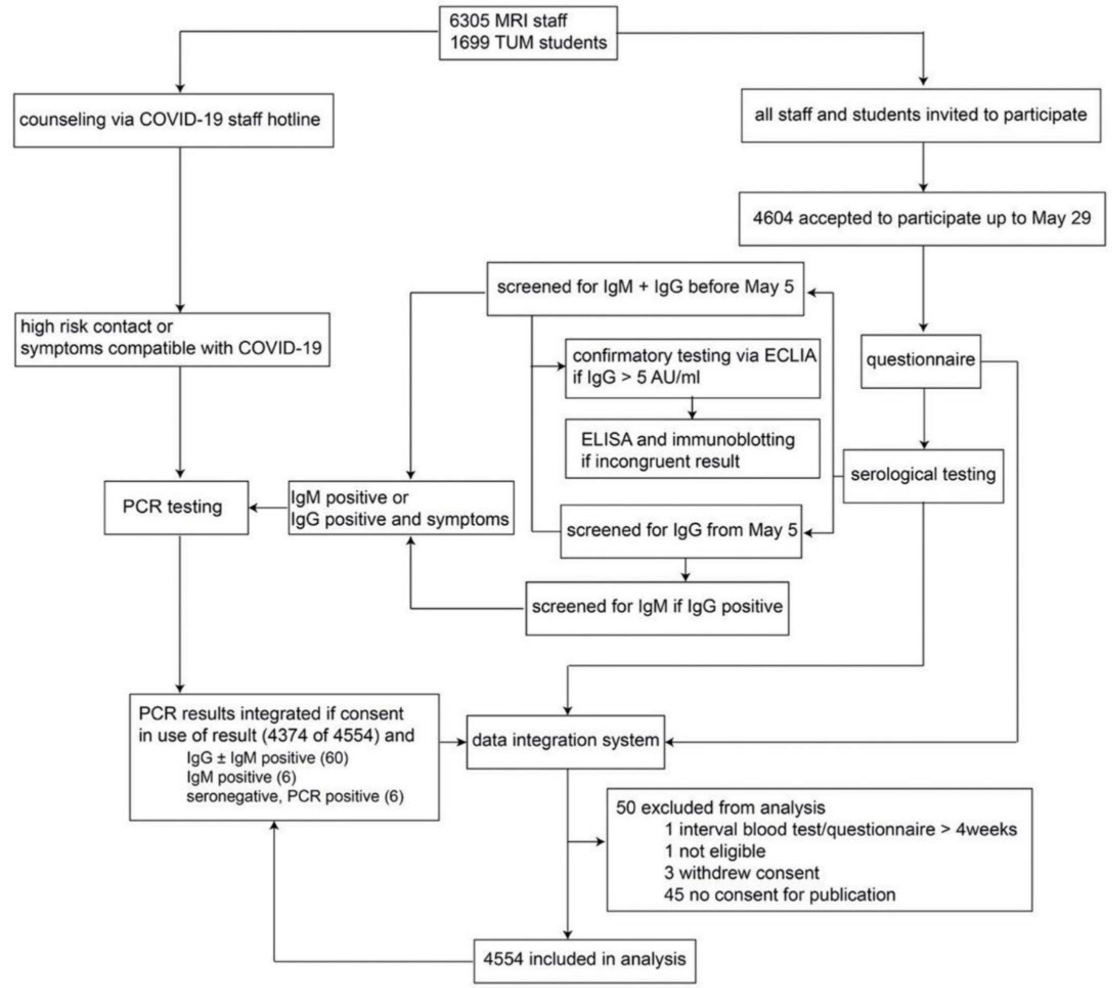

Appendix Figure 1. Study flowchart illustrating the testing algorithm and included results. CLIA, chemiluminescent immunoassay; ECLIA, electrochemiluminescent immunoassay; MRI, Munich rechts der Isar Hospital; TUM, Technical University Munich.

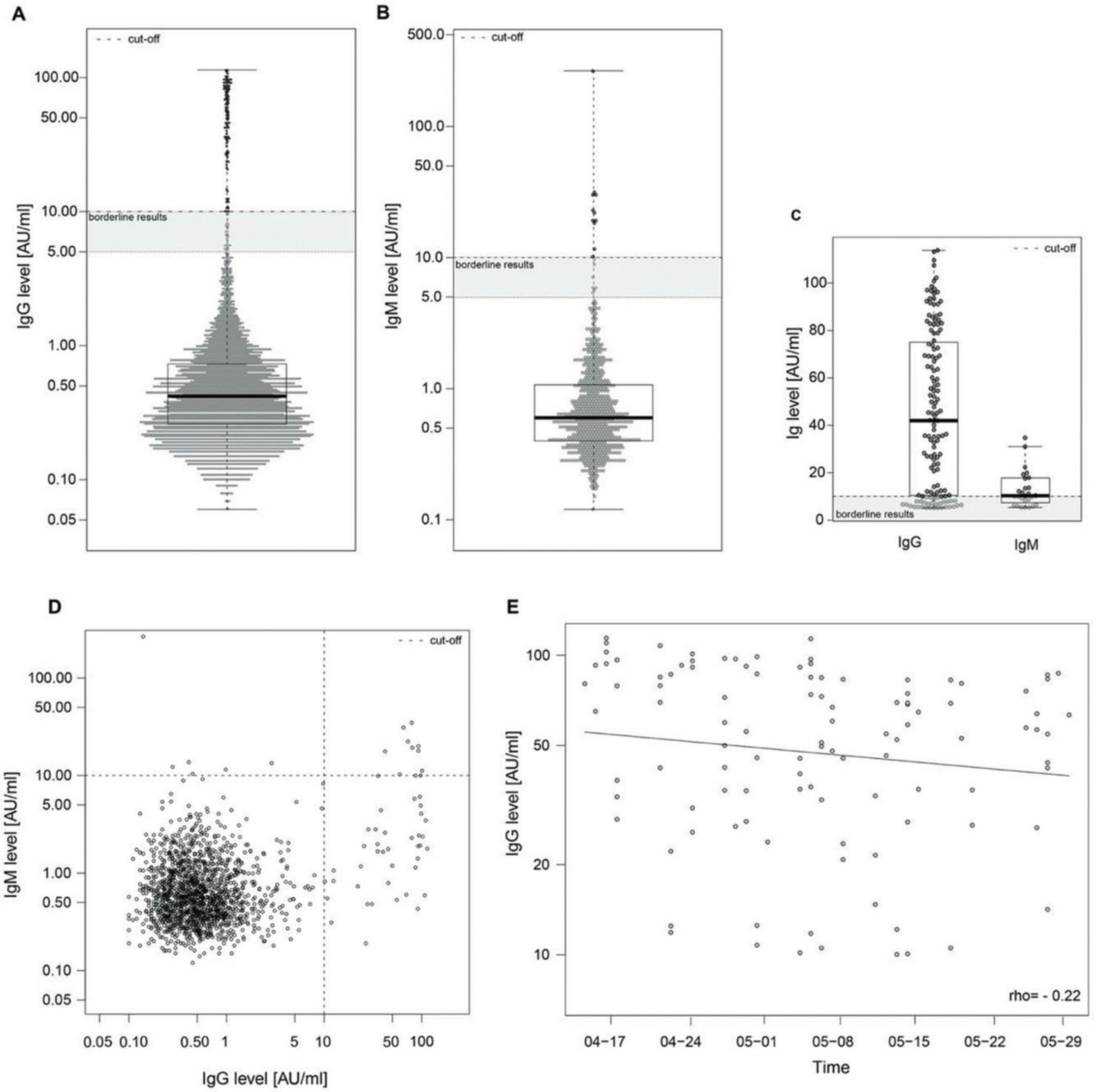

Appendix Figure 2. SARS-CoV-2 IgG and IgM levels, which were detected by using a paramagnetic particle chemiluminescent immunoassay (Shenzhen Yloh Biotech; Shenzhen, China). The cutoff was defined as ≥10 AU/mL per assay instruction, and is indicated by a dashed line. Boxplots show medians (thick middle line), as well as first and third quartiles (box boundaries), while the whiskers indicate ranges. IgG was measured in all participants (n = 4554) (A). The IgM levels of all participants tested up to May 4, 2020 (n = 1620) are depicted in (B). Thereafter, IgM was only tested in cases with positive IgG results (n = 88, data not shown). All positive IgG and IgM results (up to May 4), as well as borderline results (>5 AU/L and <10) are depicted in (C). (D) Shows the correlation of IgG and IgM levels of all participants tested for both immunoglobulins (n = 1620 = 1708). (E) IgG levels detected in seropositive persons are plotted per study day. Spearman's rank correlation coefficient was used to evaluate the association between the time point of IgG testing and the IgG titer level.

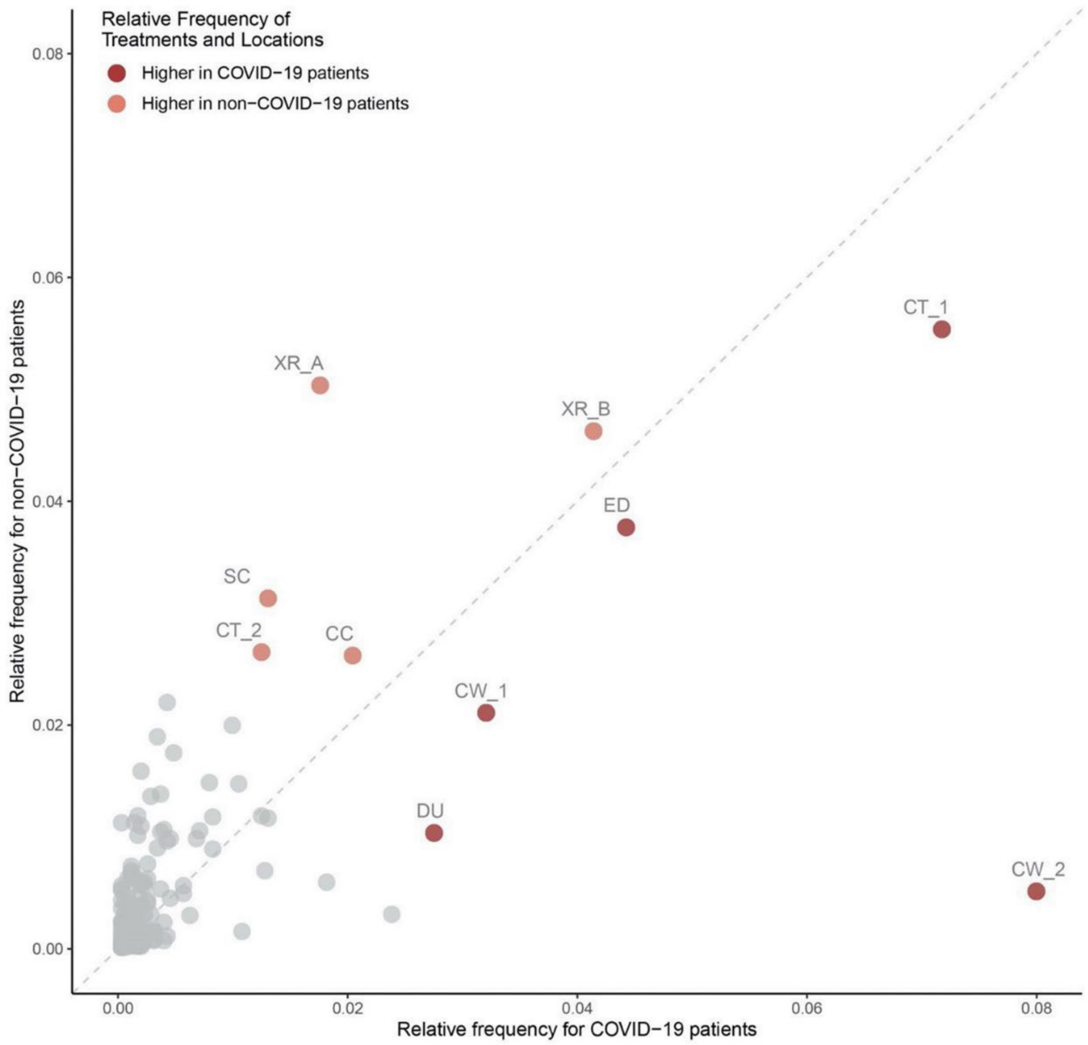

Appendix Figure 3. Relative frequency of requested diagnostics, therapies, and spatial information between patients given a diagnosis of COVID-19 and non-COVID-19 pneumonia from December 1, 2019 to June 10, 2020 normalized by each patient group. Diagram demonstrating that the diagnostic and therapeutic facilities for patients with COVID-19 and non-COVID-19 pneumonia were used differentially by the two patient groups, further limiting the possibilities of infection. CC, cardiovascular clinic; CT_1 and CT_2, spatially distinct CT scanners; CW_1, COVID-19 admission ward; CW_2, COVID-19 ward; DU, dialysis unit; ED, emergency department; SC, social counselling; XR_A and XR_B, spatially distinct x-ray units.

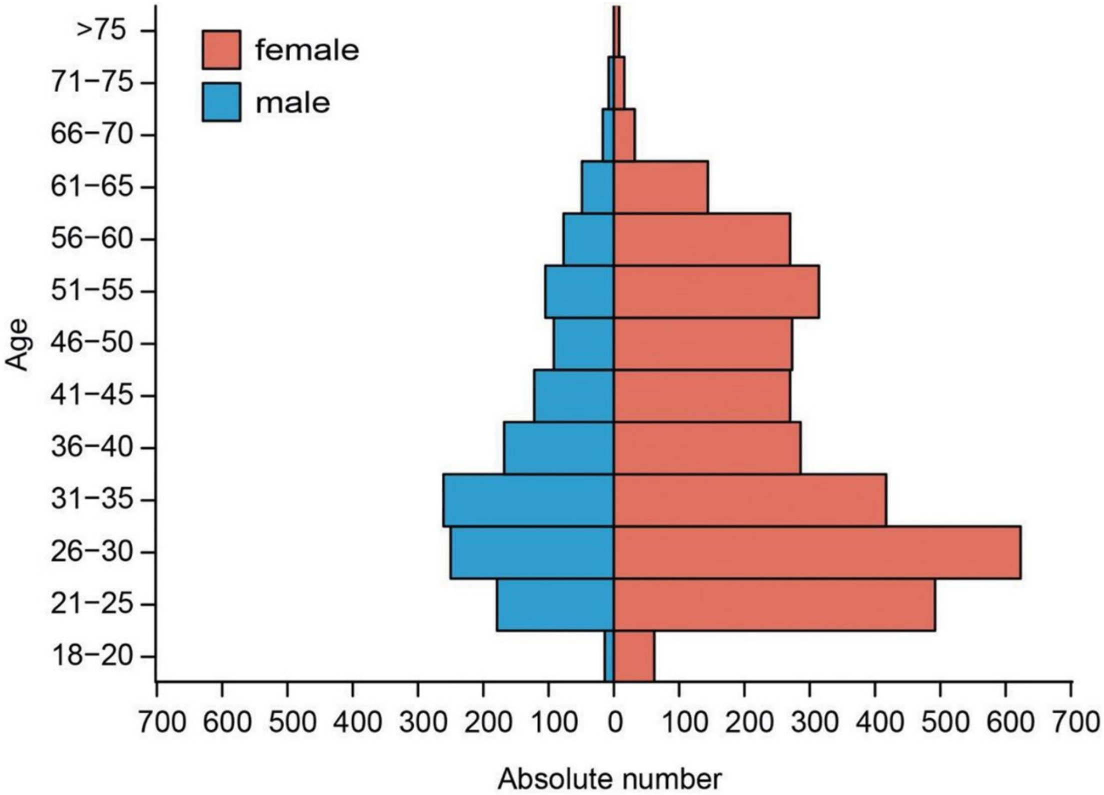

Appendix Figure 4. Age and sex distribution of study participants. Population pyramid indicates age and sex distribution of patients.

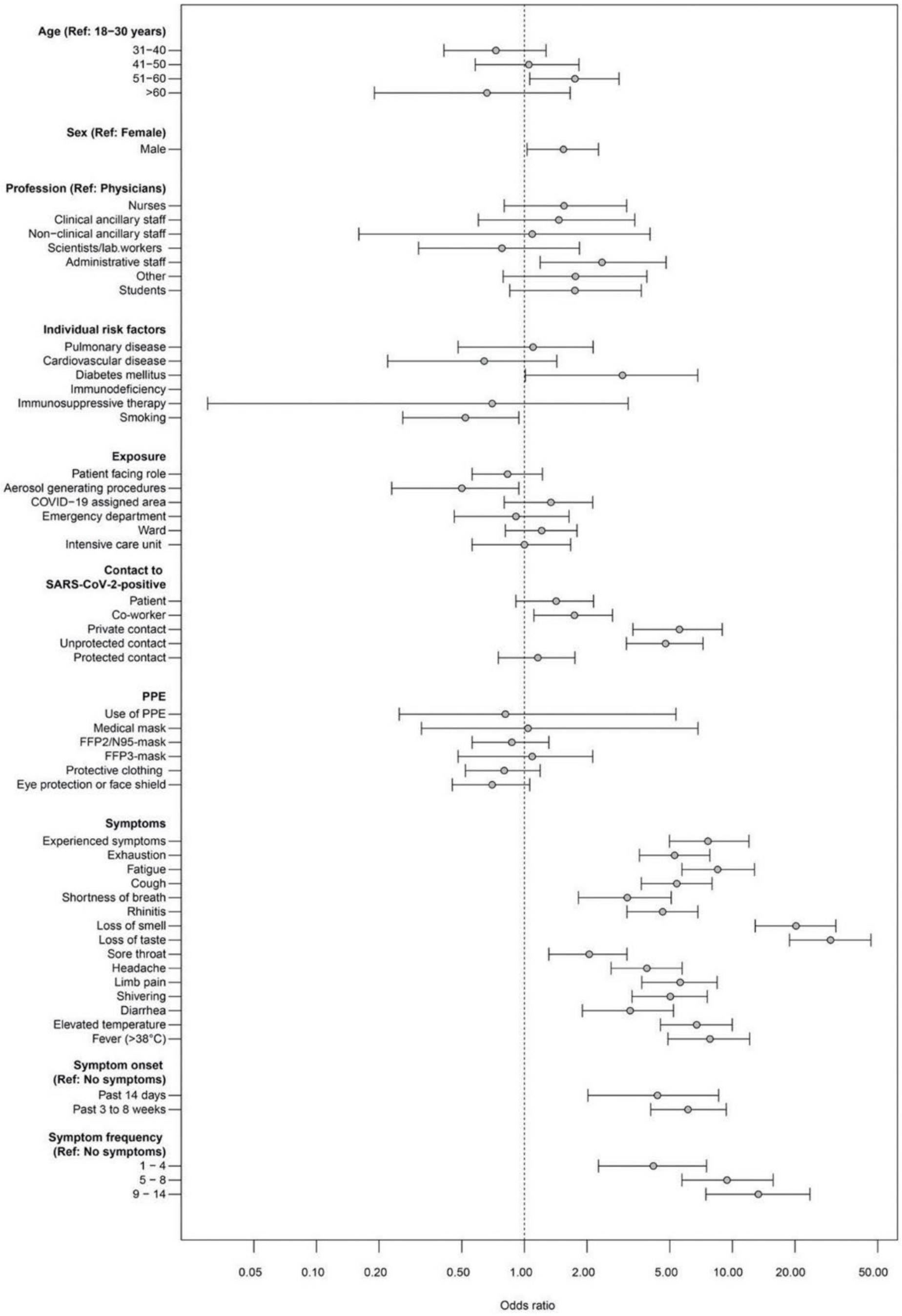

Appendix Figure 5. Graphic representation of all odds ratios for seropositivity to SARS-CoV-2 IgG represented in S1–S3 Tables. Odds ratios with exact 95% confidence intervals (mid-p intervals) are presented. FFP, filtering face piece; FFP 2, use of masks with 94% or ≥95% filter capacity for particles >0.6 μm; FFP3, use of masks with 99% filter capacity for particles >0.6 μm; Lab, laboratory, Ref, reference; PPE, personal protective equipment.

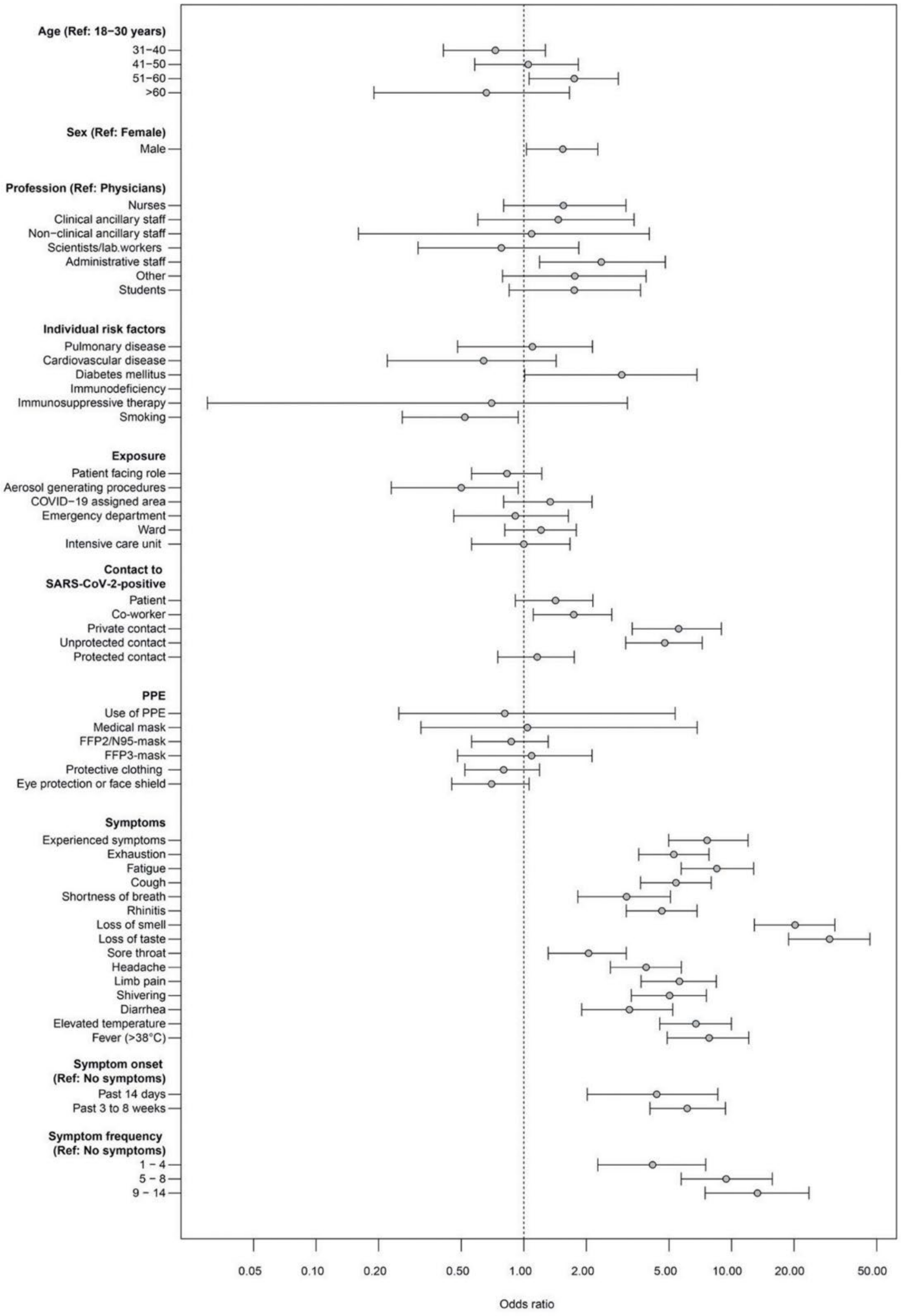

Appendix Figure 6. Distribution of SARS-CoV-2 IgG levels stratified for personal/occupational risk factors. IgG levels were compared in seropositive staff of different age (A), sex (B), and with different comorbidities or smoking status (C), reported COVID-19 contact (D), as well as occupational exposures (E) and use of distinct personal protective equipment. Boxplots show medians (thick middle line), as well as the first and third quartiles (box boundaries), while the whiskers indicate ranges. Medians and quartiles are depicted in the adjacent table. ED, emergency department; FFP, filtering face piece; ICU, intensive care unit.

A

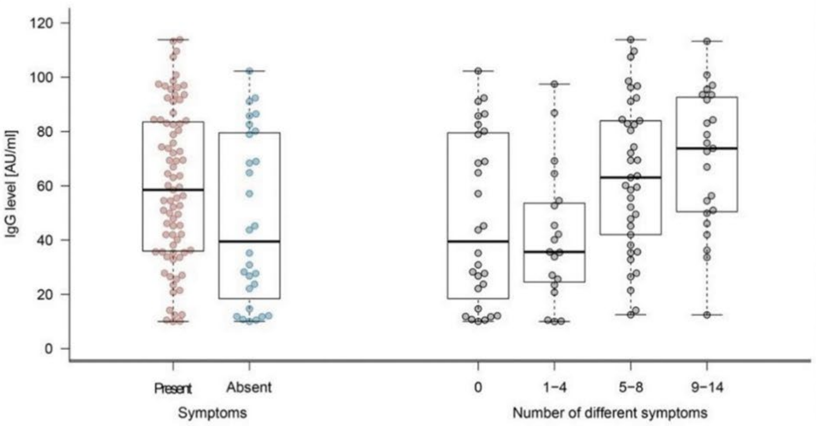

Symptoms		Overall (N=107)
Present	N	79
	Median	58.50
	Q1, Q3	35.96, 83.53
Absent	N	28
	Median	39.48
	Q1, Q3	20.26, 79.26

No. of symptoms		Overall (N=107)
0	N	28
	Median	39.48
	Q1, Q3	20.26, 79.26
1 - 4	N	19
	Median	35.63
	Q1, Q3	24.53, 53.63
5 - 8	N	37
	Median	63.04
	Q1, Q3	41.99, 83.97
9 - 14	N	23
	Median	73.79
	Q1, Q3	50.45, 92.59

B

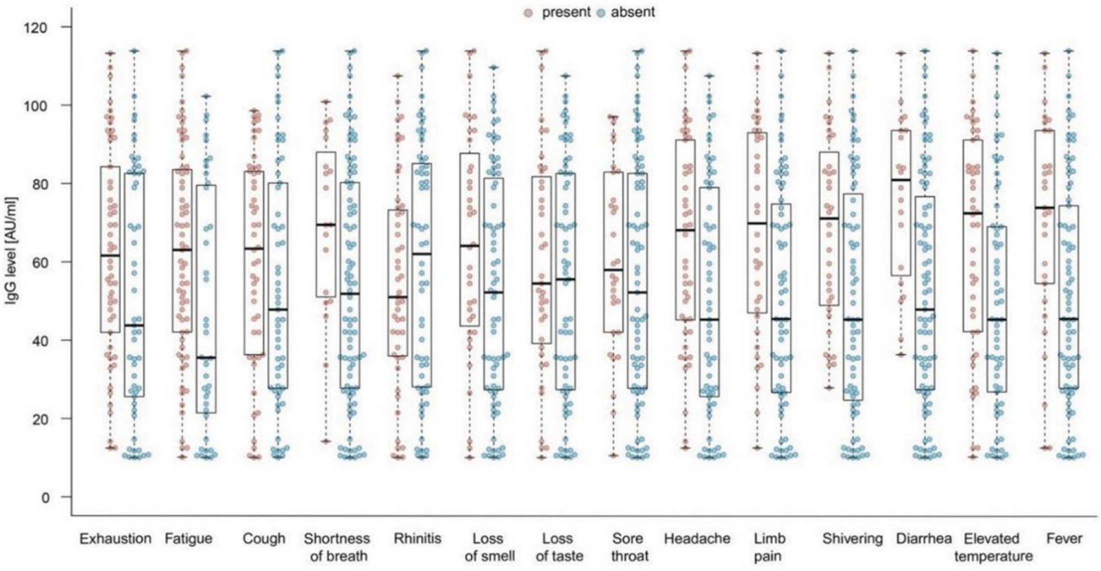

	Present	Absent
**Exhaustion**	N=54	N=53
N	54	53
Median	61.59	43.75
Q1, Q3	42.78, 83.31	25.61, 82.56
**Fatigue**	N=67	N=40
Median	63.04	35.53
Q1, Q3	42.06, 83.53	21.78, 79.26
**Cough**	N=50	N=57
Median	63.36	47.8
Q1, Q3	37.70, 83.04	27.70, 80.12
**Shortness of breath**	N=19	N=88
Median	69.45	51.84
Q1, Q3	51.04, 87.98	27.78, 80.19
**Rhinitis**	N=47	N=60
Median	50.98	62.00
Q1, Q3	35.96, 73.23	28.163, 84.75
**Loss of smell**	N=36	N=71
Median	64.08	52.17
Q1, Q3	44.38, 85.98	27.37, 81.33
**Loss of taste**	N=39	N=67
Median	54.46	55.52
Q1, Q3	39.14, 81.74	27.37, 82.55

	Present	Absent
**Sore throat**	N=30	N=77
Median	57.92	52.17
Q1, Q3	43.87, 81.10	27.70, 82.56
**Headache**	N=46	N=61
Median	68.07	45.24
Q1, Q3	45.24, 89.41	25.61, 78.97
**Limb pain**	N=36	N=71
Median	69.81	45.4
Q1, Q3	47.38, 92.67	26.64, 74.77
**Shivering**	N=36	N=71
Median	71.06	45.24
Q1, Q3	49.38, 86.13	24.680, 77.36
**Diarrhea**	N=20	N=87
Median	80.88	47.8
Q1, Q3	57.49, 93.51	27.370, 76.64
**Elevated temperature**	N=46	N=61
Median	72.39	45.24
Q1, Q3	43.13, 89.41	26.78, 68.97
**Fever**	N=29	N=77
Median	73.79	45.4
Q1, Q3	54.46, 93.50	27.70, 74.31

Appendix Figure 7. SARS-CoV-2 IgG levels and symptoms. Distribution of antibodies stratified for symptom frequency (A) and character (B). Boxplots show medians (thick middle line), as well as first and third quartiles (box boundaries). Values are annotated in the adjacent table. Whiskers indicate ranges. Q, quartile.

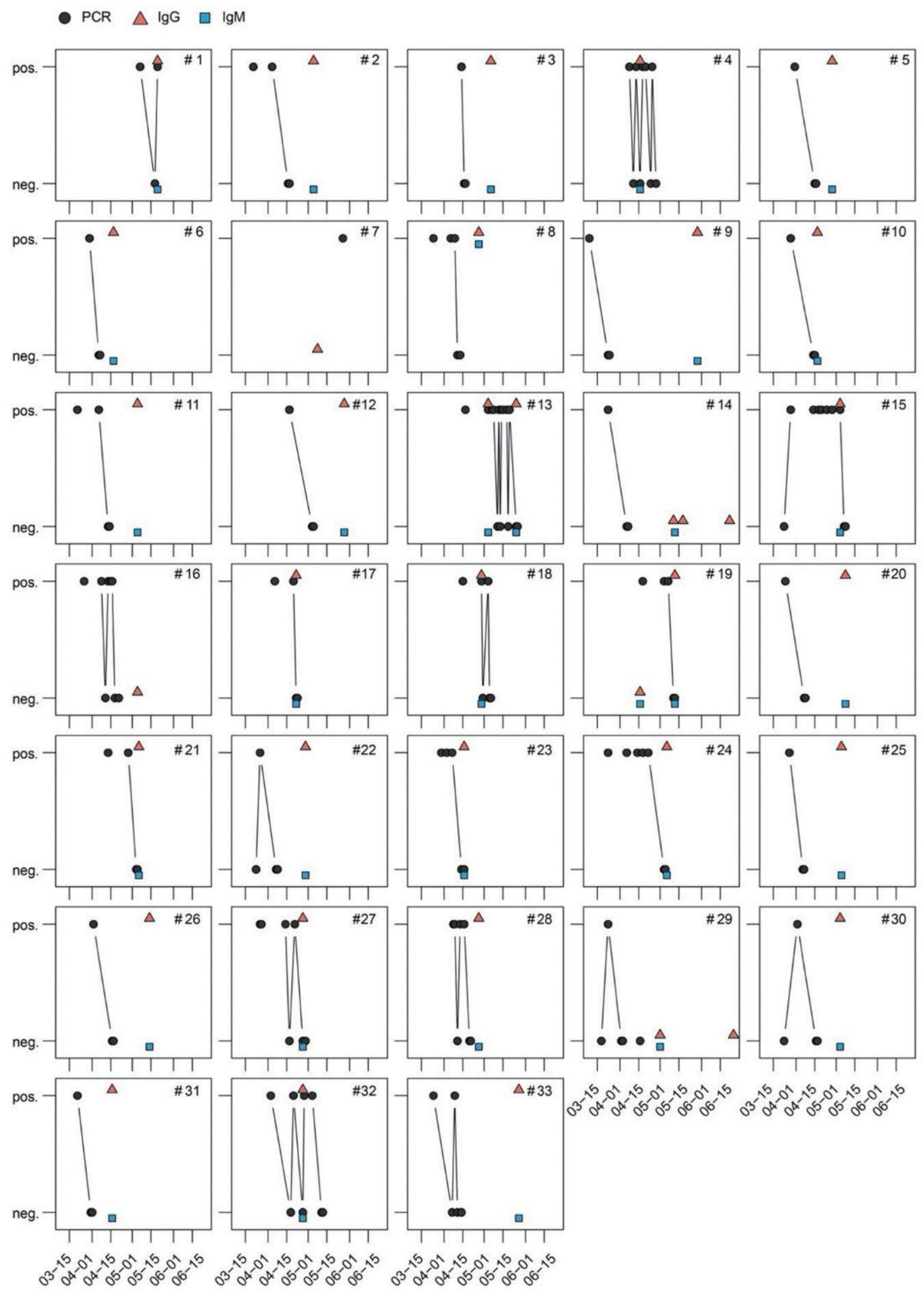

Appendix Figure 8. Time course of SARS-CoV-2 antibody and PCR test results in 33 employees. PCR test results were available for 33 employees (numbered consecutively) who had tested positive for SARS-CoV-2 by PCR at least once. Plots show all SARS-CoV-2 IgG and IgM results, as well as PCR tests performed before June 15, 2020, for these patients. Five patients (Nos. 7, 14, 16, 19, and 29) tested negative for IgG at the time of the serosurvey. Seroconversion could be detected for patient no. 19 at follow-up assessment. Pos, positive; Neg, negative.
